# The intraviral protein-protein interaction of SARS-CoV-2 reveals the key role of N protein in virus-like particle assembly

**DOI:** 10.7150/ijbs.64977

**Published:** 2021-09-07

**Authors:** Minghai Chen, Chuang Yan, Fujun Qin, Luping Zheng, Xian-En Zhang

**Affiliations:** 1 CAS Key Laboratory of Quantitative Engineering Biology, Shenzhen Institute of Synthetic Biology, Shenzhen Institutes of Advanced Technology, Chinese Academy of Sciences, Shenzhen 518055, China.; 2 Faculty of Synthetic Biology, Shenzhen Institutes of Advanced Technology, Chinese Academy of Sciences, Shenzhen 518055, China.; 3 National Laboratory of Biomacromolecules, CAS Center for Excellence in Biomacromolecules, Institute of Biophysics, Chinese Academy of Sciences, Beijing 100101, China.

**Keywords:** SARS-CoV-2, interactome, nucleocapsid protein, BiFC, VLPs

## Abstract

Intraviral protein-protein interactions (PPIs) of SARS-CoV-2 in host cells may provide useful information for deep understanding of virology of SARS-CoV-2. In this study, 22 of 55 interactions of the structural and accessory proteins of SARS-CoV-2 were identified by biomolecular fluorescence complementation (BiFC) assay. The nucleocapsid (N) protein was found to have the most interactions among the structural and accessory proteins of SARS-CoV-2, and also specifically interacted with the putative packaging signal (PS) of SARS-CoV-2. We also demonstrated that the PS core containing PS576 RNA bears a functional PS, important for the assembly of the viral RNA into virus like particles (VLPs), and the packaging of SARS-CoV-2 RNA was N dependent.

## Introduction

SARS-CoV-2 is a positive-stranded RNA virus with a 30 kb genome 1, 2. The first two-thirds of the SARS-CoV-2 viral RNA genome, the ORF1a/b region, encodes 16 non-structural proteins. The remaining parts of the viral genome encode several accessory proteins and four structural proteins, including the spike (S) glycoprotein, envelope (E), matrix (M) and nucleocapsid (N) proteins. These four structural proteins are important for maintaining the structural integrity of the enveloped SARS-CoV-2 virion 3-6. Previous studies showed that the intraviral protein-protein interactions (PPIs) of SARS-CoV play pivotal roles in many processes during the viral infection cycle, including the formation of virus replication complexes, assembly of virions and coordinated functions between different viral proteins 7-9. However, very little is known about the PPIs of SARS-CoV-2 viral proteins. PPI analysis of SARS-CoV-2 is important for understanding viral protein functions and to elucidate the related molecular mechanisms for assembly and formation of virions.

Recently, evaluation of the mechanisms of viral budding and entry, and assessment of drug inhibitors under BSL-2 conditions using SARS-CoV-2 virus-like particles (VLPs) have been reported 10. However, the exact assembly mechanisms of these VLPs remains unknown. In addition, viral packaging signal (PS) contributes to the assembly of SARS-CoV RNA through interactions with N protein 11, yet this remains poorly understood for SARS-CoV-2. In this study, 22 of 55 interactions among the structural and accessory proteins of SARS-CoV-2 were identified with the Venus-based bimolecular fluorescence complementation (BiFC) assay. Of the identified proteins, N protein was found to have the most interactions, and also specifically interacted with the putative PS of SARS-CoV-2. We also revealed that the PS core containing PS576 RNA bears a functional PS in SARS-CoV-2, which is important for assembly of the viral RNA into VLPs. Furthermore, we demonstrated that packaging of SARS-CoV-2 RNA was N protein dependent.

## Methods

### Plasmid construction

To identify the interactions of the SARS-CoV-2 structural and accessory proteins, a Venus-based BiFC system was used in this study 12. VN154, representing amino acids (aa) 1-154 of fluorescent protein Venus, was PCR amplified from plasmid pcDNA3.1-Venus (Sangon, Shanghai, China) and then inserted into a pcDNA3.1 vector using restriction enzymes *Not*I and *Xho*I. VC155, representing aa 155-238 of Venus, was PCR amplified from plasmid pcDNA3.1-Venus (Sangon) and then inserted into pcDNA3.1 vector using restriction enzymes *Nhe*I and *Kpn*I. The structural and accessory proteins of SARS-CoV-2 including S, E, M, N, 3a, 6, 7a, 7b, 8 and 10, were PCR amplified from plasmid pUC57-Genome (Sangon) and then inserted into the pcDNA3.1-VN154 vector to construct the plasmids pS-VN154, pE-VN154, pM-VN154, pN-VN154, p3a-VN154, p6-VN154, p7a-VN154, p7b-VN154, p8-VN154 and p10-VN154 using restriction enzymes *Nhe*I and *Kpn*I. Similarly, S, E, M, 3a, 6, 7a, 7b, 8 and 10 were PCR amplified from plasmid pUC57-Genome and inserted into the pcDNA3.1-VC155 vector to construct the plasmids pVC155-S, pVC155-E, pVC155-M, pVC155-3a, pVC155-6, pVC155-7a, pVC155-7b, pVC155-8 and pVC155-10 using restriction enzymes *Kpn*I and *Xho*I. N was PCR amplified from plasmid pUC57-Genome and inserted into the pcDNA3.1-VC155 vector to construct the plasmid pVC155-N using restriction enzymes *BamH*I and *Not*I. Plasmids pN(1-246)-VN154, pN(247-419)-VN154, pVC155-N(1-246) and pVC155-N(247-419) were generated by replacing pN-VN154 or pVC155-N with N(1-246) or N(247-419), respectively.

For the generation of plasmids pcDNA3.1-S and pcDNA3.1-Flag-E-T2A-Flag-M-T2A-N or pcDNA3.1-Flag-E-T2A-Flag-M, S was PCR amplified and inserted into the pcDNA3.1 vector using restriction enzymes *Nhe*I and *Kpn*I. Flag tag and T2A sequences were included in the E-M-N or E-M sequences and then inserted into the pcDNA3.1 vector using restriction enzymes *Nhe*I and *Kpn*I. Plasmid pEGFP-N1-PS576 was developed by amplifying the putative PS of SARS-CoV-2 genomic RNA from nucleotide (nt) 19786-20361 13 and inserted into the 3′ noncoding region of the EGFP gene. To generate the plasmid pN-iRN123, N was PCR amplified and then used to replace YBX1 in plasmid pYBX1-iRN123 14. pECFP-ms2-PS576 was generated by replacing HOTAIR in the plasmid pECFP-ms2-HOTAIR 14. Plasmids pN(1-246)-iRN123 and pN(247-419)-iRN123 were also constructed by replacing N in the plasmid pN-iRN123.

The primers used in this work are listed in **Table S1**. All of the sequences of the constructs were confirmed by DNA sequencing.

### Cell culture

HEK 293T and human angiotensin-converting enzyme 2 expressed 293T (hACE2-293T) cells were maintained at 37 °C with 5% CO_2_ in Dulbecco's modified Eagle's medium (DMEM) supplemented with 10% heat-inactivated fetal bovine serum (FBS) plus 100 U/ml penicillin and 100 μg/ml streptomycin in a humidified incubator.

### Transfection and production of SARS-CoV-2 VLPs

To identify PPIs, HEK 293T cells were seeded the day before transfection on 35 mm glass-bottomed wells. Two micrograms of each plasmid were transfected using Lipofectamine 2000 (Invitrogen, Carlsbad, CA). Transfected cells were then cultured at 37 °C (5% CO_2_) for 24 h before imaging. For production of SARS-CoV-2 VLPs, HEK 293T cells were seeded the day before transfection in 10 cm culture dishes. Ten micrograms of each of the structural protein-encoding plasmids pcDNA3.1-S and pcDNA3.1-Flag-E-T2A-Flag-M-T2A-N or pcDNA3.1-Flag-E-T2A-Flag-M were co-transfected using Lipofectamine 2000. After 6 h transfection, the transfection medium was removed and 10 ml of DMEM containing 10% heat-inactivated FBS was added. The transfected cells were then incubated at 37 °C for 2 days before harvesting. To examine the role of the PS in assembly of SARS-CoV-2, 10 μg of pEGFP-N1-PS576 was co-transfected with the plasmids encoding the four structural proteins, pcDNA3.1-S and pcDNA3.1-Flag-E-T2A-Flag-M-T2A-N, into HEK 293 T cells, generating VLP (EGFP-PS576). For generation of VLPdN, co-transfection experiments were performed with 10 μg of pcDNA3.1-Flag-E-T2A-Flag-M, pcDNA3.1-S and the PS plasmid pEGFP-N1-PS576.

### Fluorescence imaging

Cells were imaged with a Nikon TiE inverted microscope using a 60× oil immersion objective lens. Venus and EGFP fluorescence were excited using a 488 nm laser, while iRFP fluorescence was excited with a 640 nm laser. Hoechst 33342 was used to stain cell nuclei and was excited using a 405 nm laser.

### Harvest and purification of SARS-CoV-2 VLPs

Harvest and purification of SARS-CoV-2 VLPs was performed as described previously 15, with minor modifications. Briefly, the supernatant was collected 48 h post-transfection and centrifuged at 5,000 × g for 10 min to remove large cellular debris. To further remove unwanted debris, the supernatant was filtered through a 0.45 µm filter (Millipore, Burlington, MA). The supernatant was then layered over a 20% sucrose buffer (20% sucrose, PBS, pH 7.4) and centrifuged at 100,000 g in an P32ST rotor (Eppendorf Himac Technologies, Hamburg, Germany) for 4 h at 4 °C. The virion pellet was resuspended in PBS and stored at -80 °C.

### Transmission electron microscopy

Carbon-coated copper grids were placed on the samples for 10 min, and the redundant liquid was removed by filter paper. The grids were then negatively stained with 10 µl of 1% phosphotungstic acid (PTA, pH 7.0) for 60 s at room temperature. Prepared copper grids were examined by FEI Tecnai G2 F20 S-TWIN (FEI Company, Hillsboro, OR) transmission electron microscopy (TEM). *Autographa californica* multiple nucleopolyhedrovirus (AcMNPV) was kindly provided by Prof. Xiulian Sun (Wuhan Institute of Virology, CAS) was used as an internal control when making comparisons.

### Western blot analysis

Protein lysates prepared from VLPs were subjected to sodium dodecyl sulfate-polyacrylamide gel electrophoresis. Proteins were then transferred onto polyvinylidene fluoride membranes. After blocking at 37 °C for 2 h with PBS supplemented with 5% (w/v) skim milk, the membranes were incubated with specific antibodies (Abcam, Cambridge, UK) at 4 °C overnight. The membranes were then incubated with horseradish peroxidase-conjugated goat anti-mouse IgG (Abcam) or goat anti-rabbit IgG (Abcam) at 37 °C for 2 h, and bands were detected using a chemiluminescence detection system (BioRad, Hercules, CA).

### Reverse transcription PCR analysis

To detect the expression of the structural proteins and PS576 of SARS-CoV-2, transfected cells were collected and the RNA was purified with an RNA extraction kit (Magen, Guangzhou, China). Reverse transcription was then conducted to acquire the cDNA template using a reverse transcription kit (Vazyme, Nanjing, China) according to the manufacturer's instructions. Specific primers (**Table S1**) were then used to amplify the structural protein genes and PS576 sequence. GAPDH was used as the internal control. Similar procedure was conducted to detect the EGFP and PS576 in VLP (EGFP-PS576) or VLPdN (EGFP-PS576) virions.

## Results

### Identification of SARS-CoV-2 intraviral PPIs in live cells by BiFC assay

**Figure 1:**
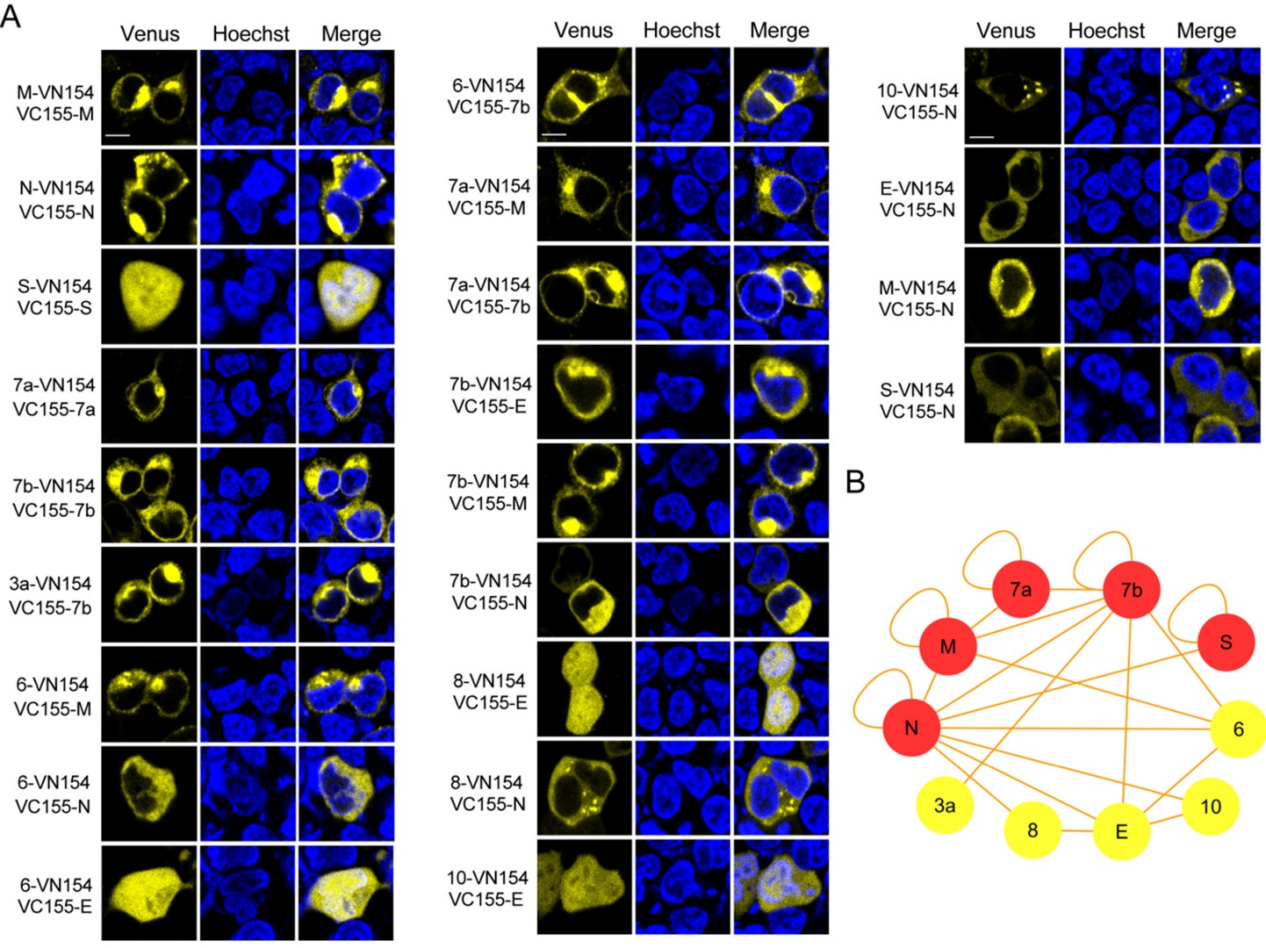
**Identification of the structural and accessory protein interactions of SARS-CoV-2 by BiFC assay.** (A) Twenty-two interactions among the structural and accessory proteins of SARS-CoV-2 were visualized using a split-Venus based BiFC assay. (B) PPI analysis for the identified interacting proteins in (A). A PPI network of interacting proteins was constructed using Cytoscape software. Red nodes in the network indicate proteins undergoing self-interaction. Nuclei were stained with Hoechst 33342. Scale bars, 10 µm. Three repeats were conducted during the screening experiment.

We used the recently developed Venus fluorescent protein based BiFC system, which has a bright complementary fluorescence intensity and matures at the physiological conditions to identify the intraviral PPIs of SARS-CoV-2 12. Considering the important roles that SARS-CoV-2 structural and accessory proteins might play in the formation of virions, we amplified the ORFs for structural and accessory proteins, and inserted these into the Venus-based BiFC vectors separately. The Venus-based BiFC assay was based on the reconstruction of the Venus fluorescent protein from its two nonfluorescent splits fused with two interacting proteins (**Figure S1**). All the primers used for amplification were designed by inserting appropriate restriction sites and are shown in **Table S1**. In total, 55 interaction combinations between the structural and accessory proteins of SARS-CoV-2 were tested with the BiFC assay. The BiFC plasmids carrying structural and accessory genes were then co-transfected into HEK 293T cells and imaged to identify interactions. As a result, 22 interactions were detected using the BiFC assay (**Figure 1A**), while the remaining 33 interaction combinations did not produce any detectable Venus complementary fluorescence (**Figure S2**). Fluorescent spectroscopy analysis results further confirmed those interactions identified through BiFC assay (**Figure S3**). Detailed results of the interactions are listed in **Table 1**. Self-interactions were observed in M, N, S, 7a and 7b proteins, suggesting that these proteins could form dimeric or multimeric complexes by interacting with themselves. N protein was found to have the most interactions, indicating that it might play important roles in viral assembly or release processes.

Considering that most of the observed interactions of N protein were among the structural and accessory proteins of SARS-CoV-2, we selected N protein for further investigation. We generated two split region fragments based on the structure of N protein to reveal the regions of this protein that mediated interactions with other structural proteins 13. As shown in **Figure S4**, the C-terminus of N protein mediated its dimerization, which was consistent with previous reports for SARS-CoV. Both the N-terminus and the C-terminus of N interacted with E and M, while the C-terminus of N protein mediated the interaction with S (**Figure S4B**).

### N protein plays an important role in the assembly of VLPs

**Figure 2:**
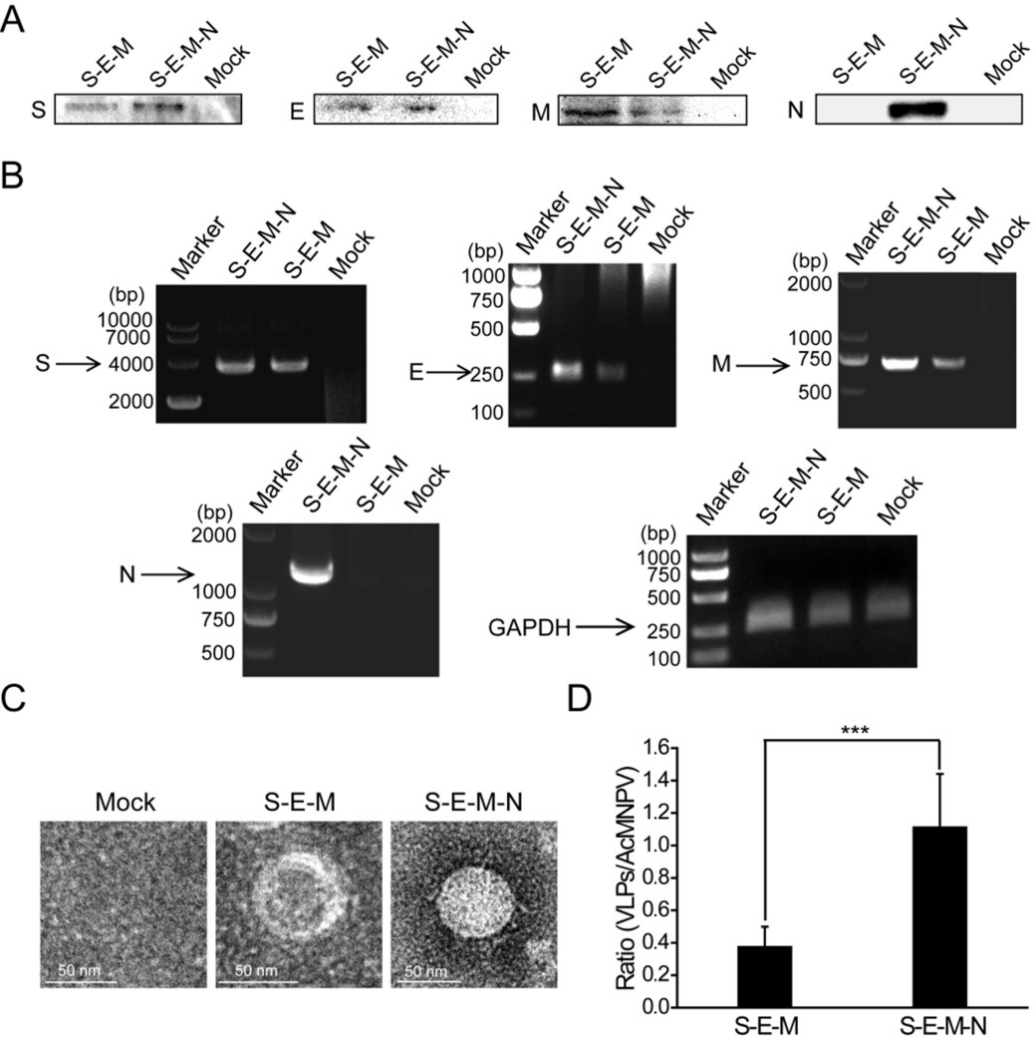
**N protein plays an important role in the assembly of VLPs.** (A) Western blot analysis of expression of the four structural proteins in the VLP fraction at 48 h post-transfection for S-E-M, S-E-M-N and mock transfected cells. (B) RT-PCR to analyze the expression of the four structural proteins in the cell lysates following transfection with the indicated combinations. (C) TEM images of VLPs formed by transfection combinations indicated. (D) Quantitative analysis of the VLPs formed from the S-E-M and S-E-M-N combinations calculated by dividing the numbers of VLPs by the numbers of AcMNPV. AcMNPV was used as the internal control. Data are given as the mean ± S.D. (n=10). Statistical significance was evaluated using a two-tailed Student's t-test. *** indicates *p*<0.01.

Following identification of the extensive interactions mediated by the N protein, we then wanted to explore the functions exerted by this protein in the assembly of VLPs. We co-transfected the plasmid encoding S with the plasmid encoding E, M and N or the plasmid encoding E and M into HEK 293T cells. At 48 h post-transfection, the culture medium and cells were harvested. The culture medium was then subjected to ultracentrifugation on a 20% sucrose cushion to isolate VLPs, and the SARS-CoV-2 structural proteins assembled into VLPs were then analyzed by western blotting. As shown in **Figure 2A**, bands for S, E and M were detected for the S-E-M plasmid combination, while bands for S, E, M and N could be detected for the S-E-M-N plasmid combination, which confirmed that the structural proteins were assembled into SARS-CoV-2 VLPs. Reverse-transcription PCR (RT-PCR) assays were also carried out to reveal expression of the corresponding structural proteins in the cell lysates (**Figure 2B**). As shown in **Figure 2C** shown, when these VLP samples were analyzed by TEM, both the S-E-M-N and S-E-M combinations formed VLPs. We then counted the observed VLPs from different combinations by mixing with the baculovirus, AcMNPV, as the internal control. As shown in **Figure S5**, we acquired AcMNPV and VLPs. The numbers of VLPs formed from the S-E-M-N combination were significantly higher than those formed from the S-E-M combination (**Figure 2D**), which indicated that N played an important role in the assembly of VLPs.

### Visualization of the interactions between N protein and the putative PS of SARS-CoV-2 by TriFC assay

**Figure 3:**
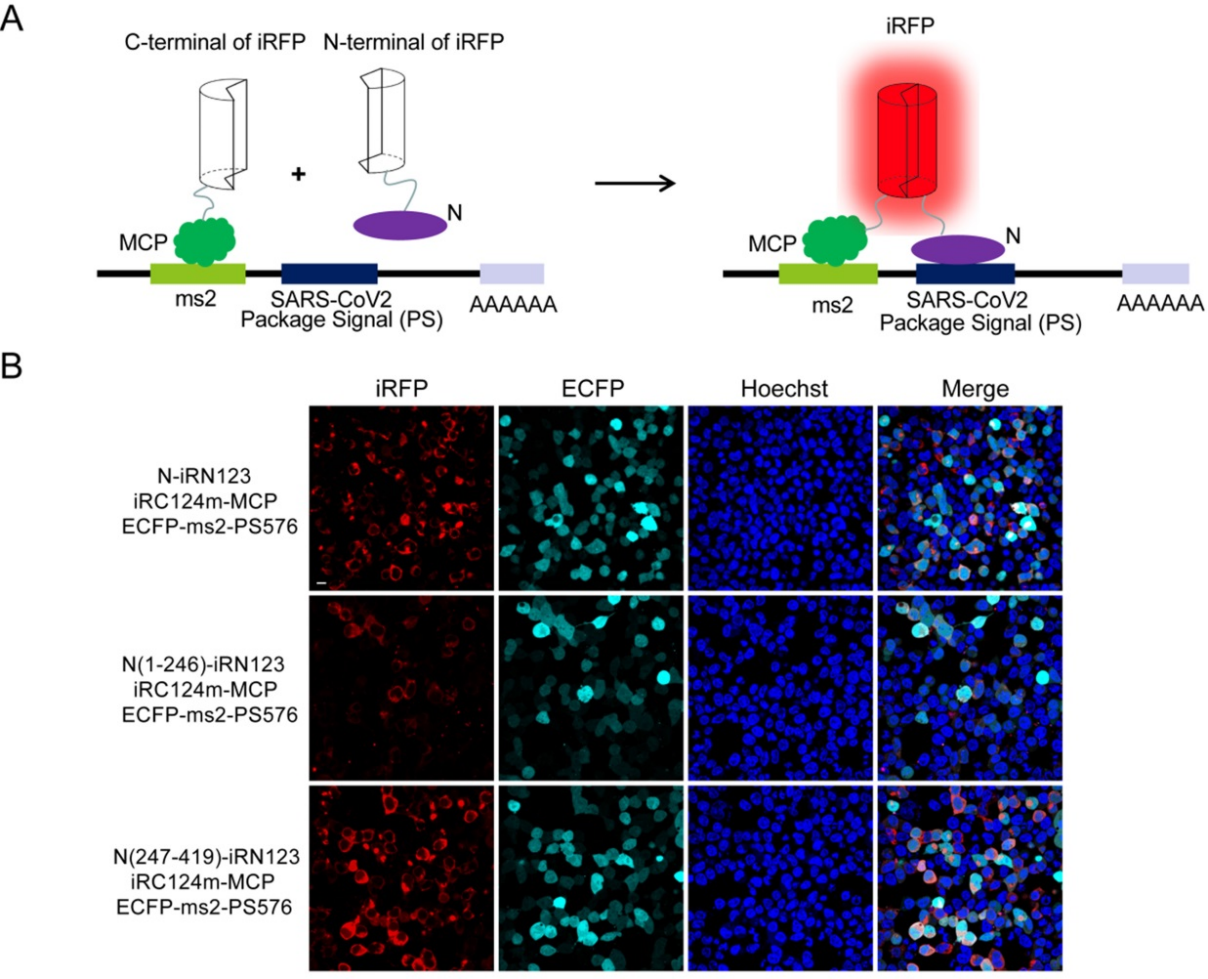
**Imaging the interactions between N and the putative PS of SARS-CoV-2 using a iRFP-based TriFC assay.** (A) The schematic principle of the iRFP-based TriFC assay. (B) Identification of the interactions and specific region mediating the interactions between N protein and PS576 using the iRFP-TriFC assay. Nuclei were stained with Hoechst 33342. Scale bar, 10 µm.

Next, we wanted to identify the interactions between N and the putative PS (PS576) of SARS-CoV-2. PS576 is a 576-nt sequence located near the end of the ORF1ab gene (nt 19786-20361) of SARS-CoV-2, which corresponding to the putative PS for SARS-CoV viral RNA 13. Here, we adopted our newly developed iRFP-based trimolecular fluorescence complementation (TriFC) system 14 to visualize RNA-protein interactions. The schematic principle of the TriFC assay is shown in **Figure 3A**. N protein was fused with the N-terminal of iRFP, PS576 was inserted into the pECFP-ms2 vector, and the constructs were then co-transfected into HEK 293T cells. As expected, red TriFC fluorescence signals could be detected in the cells co-transfected with the N and PS576 combinations (**Figure 3B**). To further identify the regions of N mediating the interaction with PS576, we replaced the full-length N protein with either the N-terminus (aa 1-246) or the C-terminus (aa 247-419) of N protein, and co-transfected the constructs into HEK 293T cells. As shown in **Figure 3B**, both N- and C-termini of the N protein interacted with PS576 but the C-terminus mainly mediated the interaction, which was consistent with the SARS-CoV N protein that has two RNA-binding sites located at the N- and C-termini of the N protein 11. Fluorescent spectroscopy analysis results further confirmed these interactions detected through TriFC assay (**Figure S6**). No observable iRFP-reconstituted fluorescence could be detected in the negative control combinations (**Figure S7**). These results verified the existence of an interaction between N and the putative PS (PS576) of SARS-CoV-2.

### Assembly of PS576 into VLPs is N-dependent

**Figure 4:**
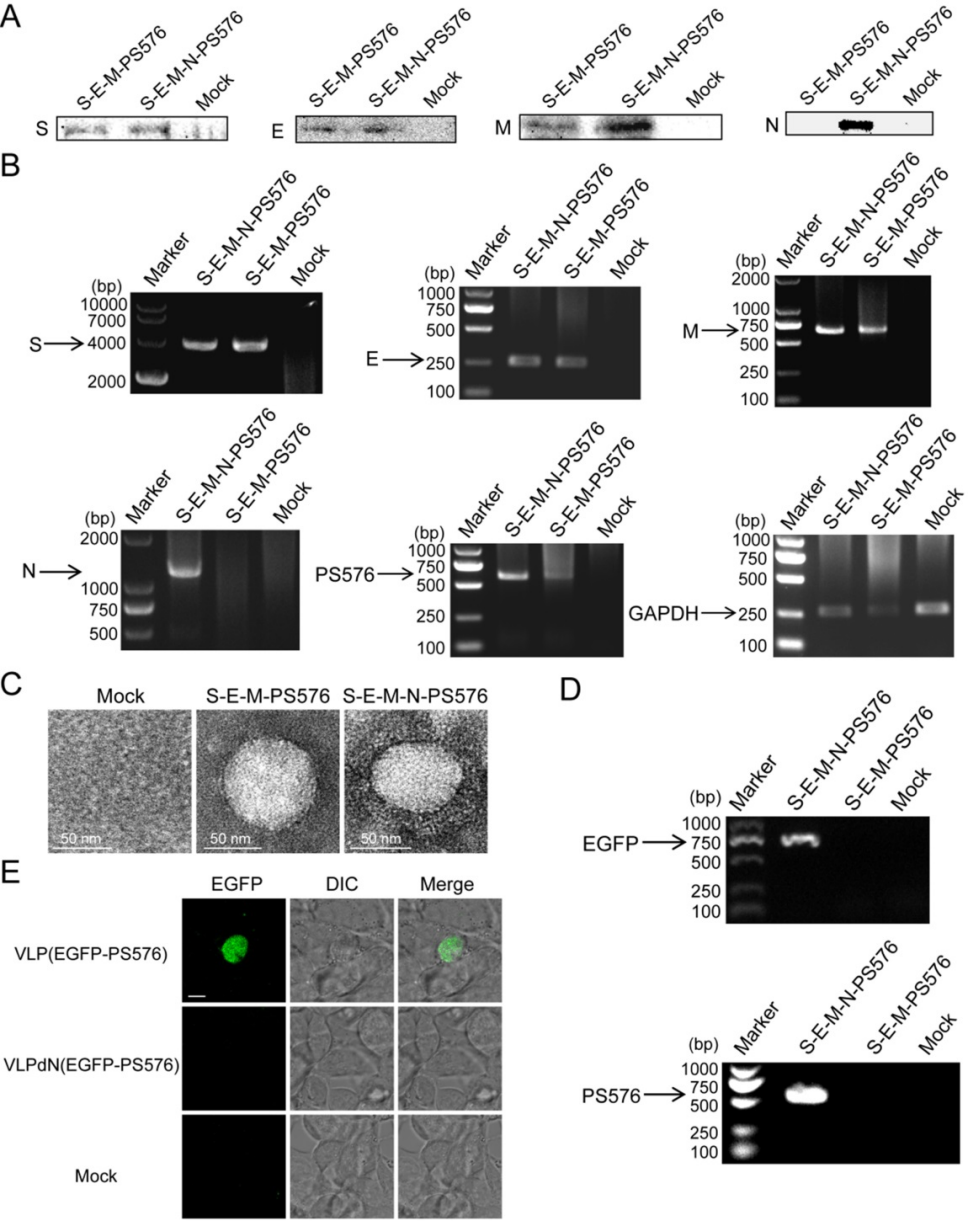
**Assembly of PS576 into VLPs was N-dependent.** (A) Western blot analysis of structural protein expression in the VLP fraction at 48 h post-transfection for S-E-M-PS576, S-E-M-N-PS576 and mock transfected cells. (B) RT-PCR analyzing the expression of the four structural proteins in cell lysates among the indicated transfection combinations. (C) TEM images of VLPs formed by transfection of indicated structural protein combinations. (D) RT-PCR analysis was conducted to detect the EGFP and PS576 sequence in virion from different combinations. (E) hACE2-293T cells were infected with either VLP(EGFP-PS576) or VLPdN(EGFP-PS576). Two days post-infection, green fluorescence was detectable in VLP(EGFP-PS576)-infected cells but not in VLPdN(EGFP-PS576)-infected cells. Mock-infected cells are shown as negative control, with undetectable fluorescence. Scale bar in (E), 10 µm.

Previous studies have shown that mouse hepatitis virus (MHV) VLPs could be assembled in an N-independent manner and that viral RNA could be packaged in the absence of the viral N protein 16. However, whether the N protein of SARS-CoV-2 is dispensable in the packaging of viral RNA remains to be further determined. To establish a culture system in which the packaging activities of PS576 RNA can be easily detected, plasmid pEGFP-N1-PS576 that represents heterologous EGFP-PS576 mRNA, comprising PS576 inserted into the 3′ noncoding region of the EGFP reporter was generated and transiently transfected into HEK 293T cells. One day after transfection, EGFP expression was detected by fluorescence microscopy, indicating successful expression of EGFP-PS576 RNA in the transfected cells (**Figure S8**). HEK 293T cells were then co-transfected with the PS plasmid pEGFP-N1-PS576 and the expressing plasmids encoding the viral structural proteins S, E and M in parallel with a combination that included the N-encoding plasmid. VLPs and VLPdN, which lacked the N protein, were harvested from the culture medium of transfected cells. Western blot analysis confirmed that the structural proteins of SARS-CoV-2 could be assembled into VLP(EGFP-PS576) and VLPdN(EGFP-PS576) (**Figure 4A**). Cells were also collected for RT-PCR analysis at 48 h post-transfection to detect expression of viral structural proteins and EGFP-PS576. As shown in **Figure 4B**, we detected RNAs of the viral structural proteins and EGFP-PS576 in the co-transfected cells. TEM also revealed that both the S-E-M-N-PS576 and S-E-M-PS576 combinations formed VLPs (**Figure 4C**).

To further investigate whether the N protein of SARS-CoV-2 was dispensable in the packaging of viral RNA, we harvested VLPs from the culture medium of transfected cells, and then detected the EGFP and PS576 in the virions through RT-PCR. As shown in **Figure 4D**, the EGFP and PS576 could be only detected in VLP(EGFP-PS576). We also used the VLPs to infect 293T cells expressing the human angiotensin-converting enzyme 2 (hACE2) receptor. Two days post-infection, green fluorescence was detectable in VLP(EGFP-PS576)-infected cells but not in VLPdN(EGFP-PS576)-infected cells (**Figure 4E**). Quantitative analysis of the EGFP signal in the infected cells also acquired consistent results (**Figure S9**). Taken together, these results indicate that the PS core containing PS576 RNA bears a functional PS and packaging of SARS-CoV-2 RNA into VLPs is N-dependent.

## Discussion

By using a Venus-based BiFC assay in living cells, 22 out of 55 interactions among the viral structural and accessory proteins of SARS-CoV-2 were identified in live cells. Our data show that the pattern of the interactome of SARS-CoV-2 was different to that of SARS-CoV 7, 8. The N protein was found to have the most interactions among the structural and accessory proteins of SARS-CoV-2, indicating its important roles in the viral life cycle. Co-transfection of plasmids encoding the viral structural proteins S, E and M into cells produced far fewer VLPs compared with co-transfection of plasmids encoding viral structural proteins S, E, M and N. These results indicate that the N protein of SARS-CoV-2 might play important roles in the assembly or release of VLPs although it is not a membrane protein. Detailed studies have shown that the C-terminus of the N protein mediated its dimerization, which was consistent with a previous report regarding SARS-CoV 17. The interactome shows that both the N- and C-termini of the N protein interacted with E and M proteins, while the C-terminus mediated the interaction with S protein. These interactions may help in the membrane protein assemble into VLPs and virus particles in some ways.

PS are defined as *cis*-acting sequences required for efficient packaging of genomic RNA during virus assembly. However, PS have not yet been identified for SARS-CoV-2 although it is known to be located in the nt 19786-20361 region of the genome 13. Here, based on our recently built iRFP-TriFC assay 14, we imaged the N protein interaction with a putative PS within the SARS-CoV-2 genomic RNA. Both the N- and C-termini of N protein could interact with PS576 (nt 19786-20361 of SARS-CoV-2, is a 576-nt sequence located near the end of the ORF1ab gene). This phenomenon is also consistent with that of N protein of SARS-CoV, which has two RNA-binding sites located at the N- and C-termini of the N protein 11. When cells expressing hACE2 receptor were infected with the harvested VLPs, green fluorescence of EGFP could only be detected in VLP(EGFP-PS576)-infected but not VLPdN(EGFP-PS576)-infected cells, indicating that the PS core containing PS576 RNA carries a functional PS and packaging of SARS-CoV-2 RNA into VLPs is N-dependent. Recently, Ju et al. reported a novel cell culture system for production of transcription and replication-competent SARS-CoV-2 VLPs (trVLP) in which N protein was also identified as important for viral genome packaging and virion assembly 18. As for the successful assembly of EGFP-PS576 into VLPs, SARS-CoV-2 VLP(EGFP-PS576) could be served as a tool for screening of inhibitors and drug evaluation. In addition, the successful use of EGFP as an indicator for the packaging of SARS-CoV-2 RNA implies a possible application in generating other heterologous mRNAs that contain the SARS-CoV-2 PS, which would be packaged into SARS-CoV-2 VLPs and expressed upon infection of specific human tissues. Thus, the SARS-CoV-2 VLPs could be used as a tissue-specific viral vector for gene therapy or drug delivery.

In conclusion, by using BiFC system, we built a live-cell interactome of the structural and accessory proteins of SARS-CoV-2, and found N protein has the most extensive interaction with other counterparts. Further experiments revealed that both assembly of VLPs and packaging of SARS-CoV-2 RNA into the VLPs are N protein-dependent. These findings are conducive to in-depth understanding of the assembly mechanism of SARS-CoV-2 and could be served as new clues for the development of anti-virus drugs.

## Supplementary Material

Supplementary figures and table.

## Figures and Tables

**Table 1:** PPIs identified by BiFC assay

	M	N	E	S	3a	6	7a	7b	8	10
M	++	++	-	-	-	+	++	++	-	-
N	++	++	+	+	-	++	-	++	++	+
E	-	++	-	-	-	++	-	++	++	+
S	-	+	-	++	-	-	-	-	-	-
3a	-	-	-	-	-	-	-	++	-	-
6	+	++	++	-	-	-	-	++	-	-
7a	++	-	-	-	-	-	+	++	-	-
7b	++	++	++	-	++	++	++	++	-	-
8	-	++	++	-	-	-	-	-	-	-
10	-	+	+	-	-	-	-	-	-	-

++, strong interaction; +, weak interaction; -, no interaction.
