# Supplementary material for: The intraviral protein-protein interaction of SARS-CoV-2 reveals the key role of N protein in virus-like particle assembly
Source: Int J Biol Sci. 2021 Sep 7;17(14):3889–97. doi: 10.7150/ijbs.64977 (PMC8495407; doi:10.7150/ijbs.64977)
Supplement: Supplementary file 1 — Supplementary figures and table. [file ijbsv17p3889s1.pdf]

Supporting Information

The intraviral protein-protein interaction of SARS-CoV-2 reveals the key role of N protein in virus-like particle assembly

Minghai Chen,[1,‡,*] Chuang Yan,[1,‡] Fujun Qin,[1] Luping Zheng,[1] Xian-En Zhang,[2, 3,*]

[1]CAS Key Laboratory of Quantitative Engineering Biology, Shenzhen Institute of Synthetic Biology, Shenzhen Institutes of Advanced Technology, Chinese Academy of Sciences, Shenzhen 518055, China

[2]Faculty of Synthetic Biology, Shenzhen Institutes of Advanced Technology, Chinese Academy of Sciences, Shenzhen 518055, China

[3]National Laboratory of Biomacromolecules, CAS Center for Excellence in Biomacromolecules, Institute of Biophysics, Chinese Academy of Sciences, Beijing 100101, China

[‡]These authors contributed equally to this work

[*]To whom correspondence should be addressed: Xian-En Zhang, Email: zhangxe@ibp.ac.cn or Minghai Chen, Email: mh.chen1@siat.ac.cn

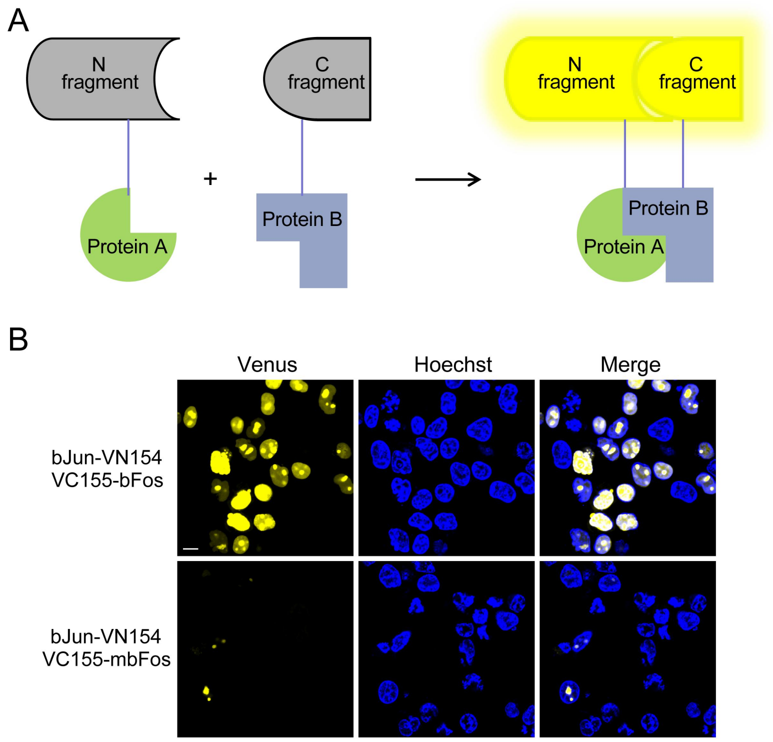

Figure S1. Imaging the PPIs with Venus-based BiFC assay. (A) The schematic principle of Venus-based BiFC assay. (B) BiFC signals (Venus channel) were detected in HEK 293T cells due to bJun-bFos interaction. bJun-mbFos interaction (bJun and mbFos do not interact) was used as the negative control. Scale bar: 10 μm.

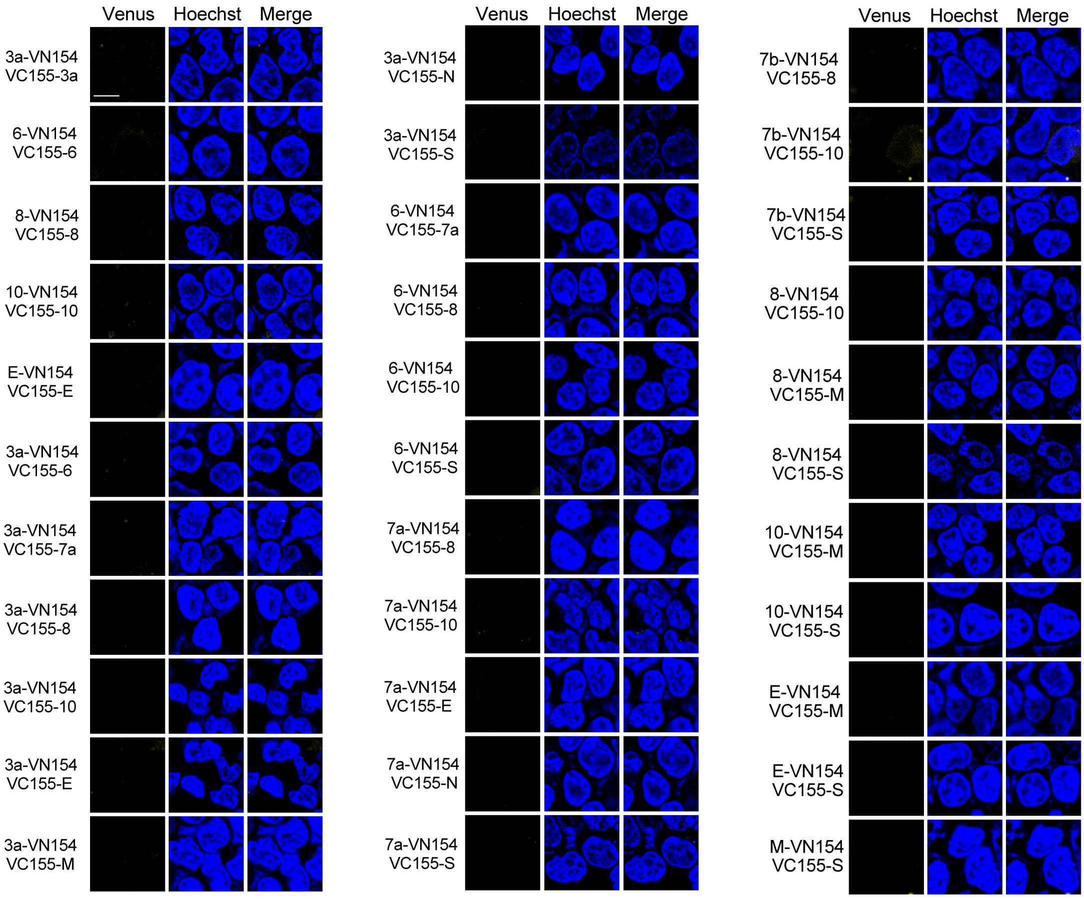

Figure S2. No observable BiFC signals were detected for the proteins that have none interactions with others among the structural and accessory proteins of SARS-CoV-2. Nuclei were stained with Hoechst 33342. Scale bar: 10 μm. Three repeats were conducted during the screening experiment.

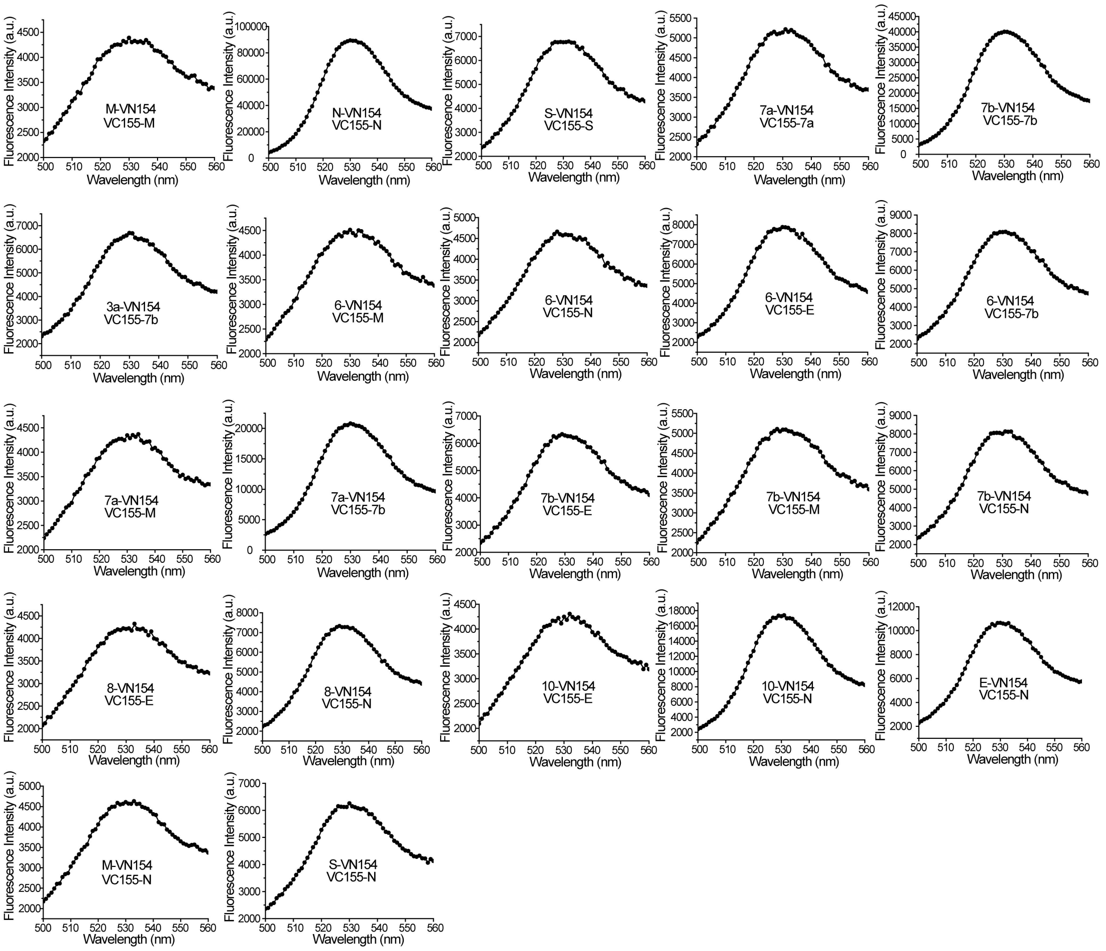

Figure S3. The emission spectra of BiFC-reconstituted Venus for the protein interactions identified in Figure 1A. The emission spectra of Venus were taken with excitation at 470 nm and collected from 500 nm to 560 nm.

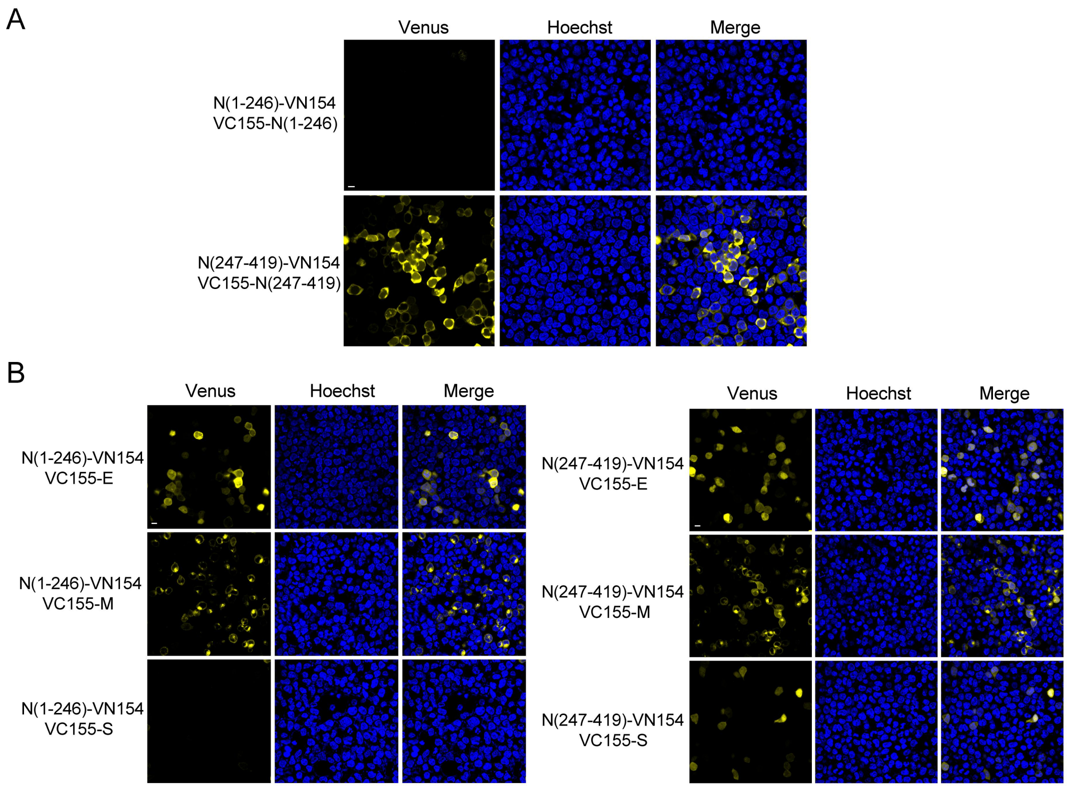

Figure S4. Identifying the specific interaction regions between N protein and other structural proteins. (A) Validation the dimeric domain of N protein by using BiFC assay. (B) Imaging the interaction regions between N protein and other structural proteins (E, M, S) by using BiFC assay. Nuclei were stained with Hoechst 33342. Scale bars: 10 μm.

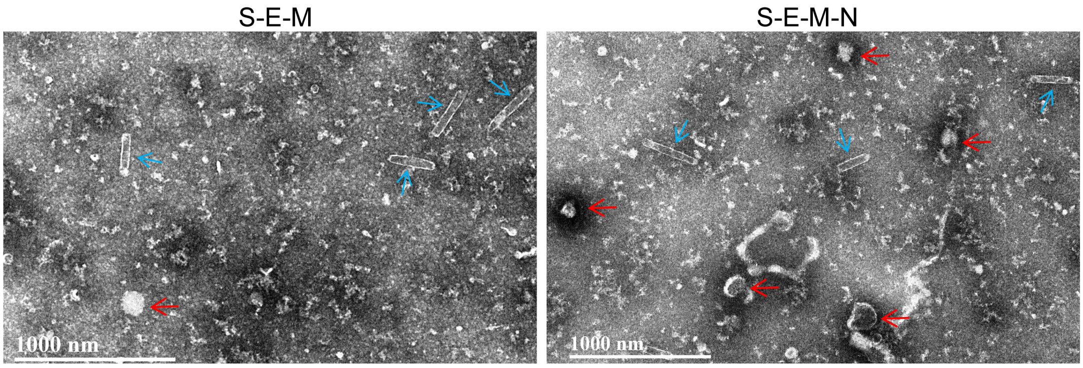

Figure S5. The TEM images of VLPs formed by S-E-M and S-E-M-N combinations, respectively. AcMNPV was used as the internal control. The red arrow indicates the SARS-CoV-2 VLPs, and the cyan arrow indicates the AcMNPV VLPs.

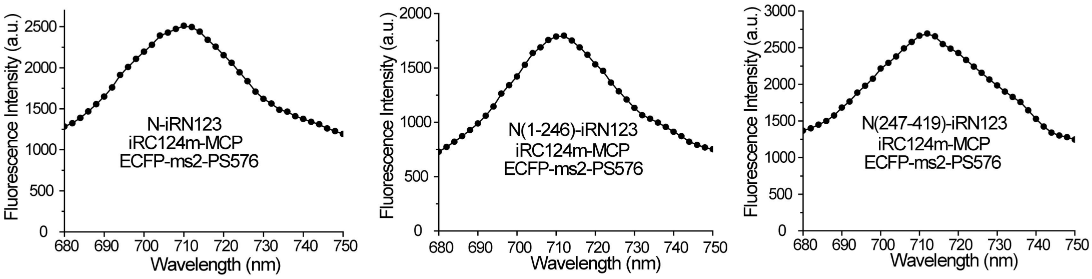

Figure S6. The emission spectra of TriFC-reconstituted iRFP for the interactions identified in Figure 3B. The emission spectra of Venus were taken with excitation at 650 nm and collected from 680 nm to 750 nm.

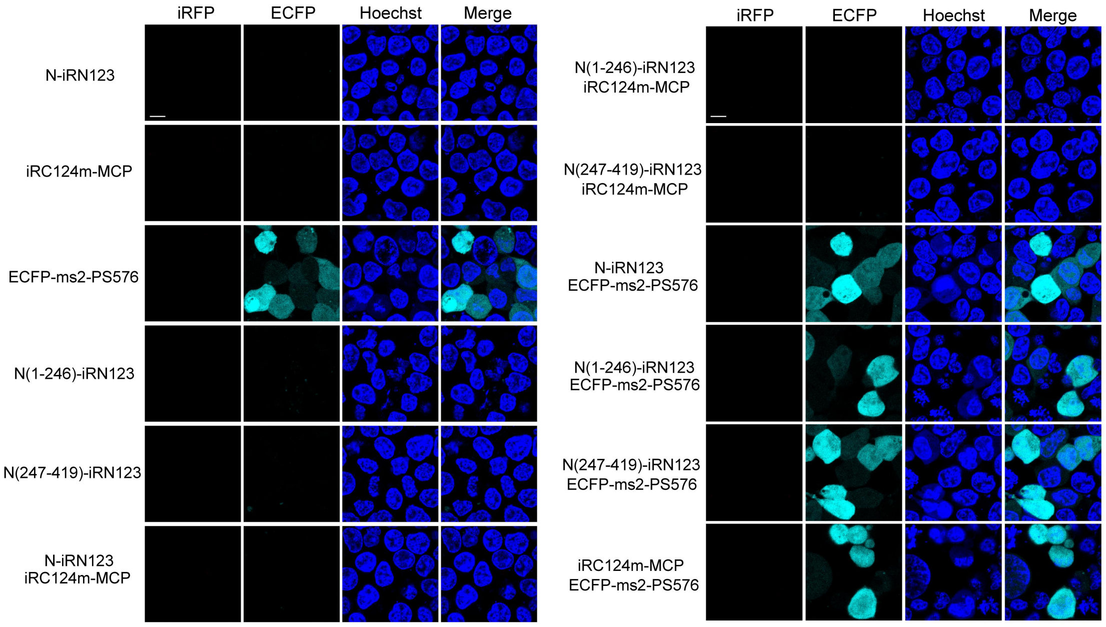

Figure S7. No observable iRFP-reconstituted signals was detected for the different negative combinations. Nuclei were stained with Hoechst 33342. Scale bars: 10 μm.

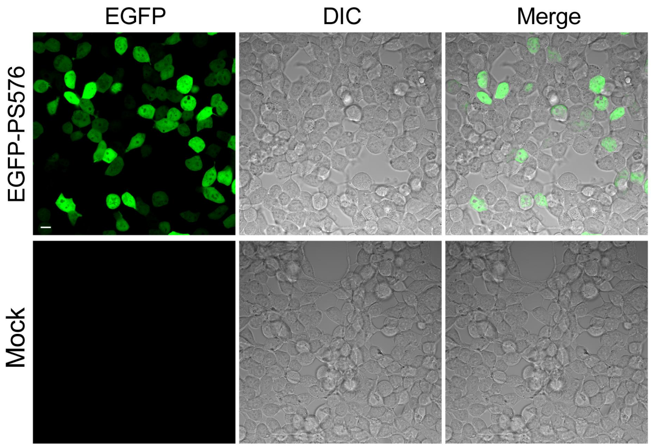

Figure S8. Expression of pEGFP-N1-PS576 in HEK 293T cells. HEK 293T cells were transfected with pEGFP-N1-PS576 bearing 576 nt of the putative packaging signal of SARS-CoV-2 inserted into the 3’ noncoding region of the EGFP gene. Green fluorescence of EGFP was visualized by confocal microscopy. Scale bar: 10 μm.

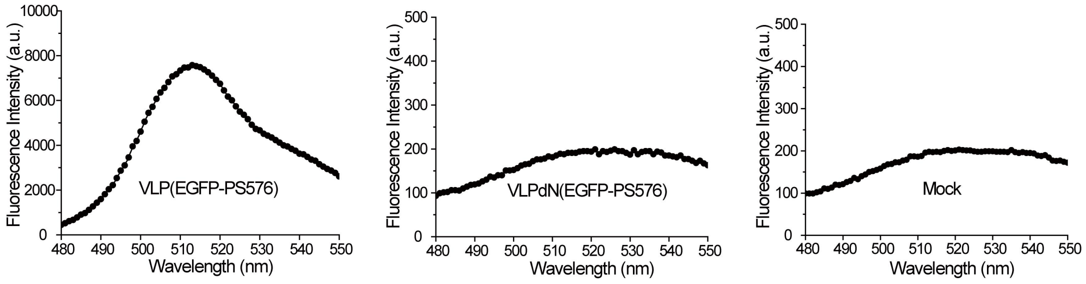

Figure S9. Quantitative analysis of the EGFP signal in the infected cells. The emission spectra of EGFP were taken with excitation at 450 nm and collected from 480 nm to 550 nm.

Table S1. Sequences of primers used in this study

Primers	Sequences (5'-3')
bJun-NheI-F	ctagctagcgccaccatgaaggcggagaggaagcgcatgagaaac
bJun-KpnI-R	ggggtaccaaacgtttgcaactgctgcgttagcatg
bFos-KpnI-F	ggggtaccggtcgtgcgcagtccatcggtcgtc
bFos-XhoI-R	ccctcgagttaacccaggtcgttcgggattttgc
mbFos-KpnI-F	ggggtaccatgggtcgtgcgcagtccatcgg
mbFos-XhoI-R	ccctcgagttaacccaggtcgttcgggattttgcac
VN154-NotI-F	atttgcggccgcatgtccaagggcgaggagctgttcacc
VN154-XhoI-R	ccctcgagttaggccgtgatgtacacgttgtgggag
VC155-NheI-F	ctagctagcgccaccatggacaagcagaagaacggcatcaag
VC155-KpnI-R	ggggtacccttgtagagctcgtccatgccg
S-NheI-F	ctagctagcgccaccatgtttgtttttcttgttttattgccactagtc
S-KpnI-R	ggggtacctgtgtaatgtaatttgactcctttgagcac
S-NotI-F	atttgcggccgcatgttcgtttttctggtgctgctgcc
S-XhoI-R	ccctcgagttaggtgtagtgcagtttcacacccttc
E-NheI-F	ctagctagcgccaccatgtactcattcgtttcggaagag
E-KpnI-R	ggggtaccgaccagaagatcaggaactctag
E-KpnI-F	ggggtaccatgtactcattcgtttcggaagag
E-XhoI-R	ccctcgagttagaccagaagatcaggaactctag
M-NheI-F	ctagctagcgccaccatggcagattccaacggtactattacc
M-KpnI-R	ggggtaccctgtacaagcaaagcaatattgtcactg
M-KpnI-F	ggggtaccatggcagattccaacggtactattacc
M-XhoI-R	ccctcgagttactgtacaagcaaagcaatattgtcactg
N-NheI-F	ctagctagcgccaccatgtctgataatggaccccaaaatcag
N-KpnI-R	ggggtaccggcctgagttgagtcagcactgctcatgg
N-BamHI-F	cgggatccatgtctgataatggaccccaaaatcag
N-NotI-R	atttgcggccgcttaggcctgagttgagtcagcactgctcatgg
3a-NheI-F	ctagctagcgccaccatggatttgtttatgagaatcttc
3a-KpnI-R	ggggtacccaaaggcacgctagtagtcgtcgtcgg
3a-KpnI-F	ggggtaccatggatttgtttatgagaatcttc
3a-XhoI-R	ccctcgagttacaaaggcacgctagtagtcgtcgtcgg
6-NheI-F	ctagctagcgccaccatgtttcatctcgttgactttcagg

6-KpnI-R	ggggtaccatcaatctccattggttgctcttc
6-KpnI-F	ggggtaccatgtttcatctcgttgactttcagg
6-XhoI-R	ccctcgagttaatcaatctccattggttgctcttc
7a-NheI-F	ctagctagcgccaccatgaaaattattcttttcttggcac
7a-KpnI-R	ggggtaccttctgtctttcttttgagtgtgaag
7a-KpnI-F	ggggtaccatgaaaattattcttttcttggcac
7a-XhoI-R	ccctcgagtcattctgtctttcttttgagtgtgaag
7b-NheI-F	ctagctagcgccaccatgattgaactttcattaattg
7b-KpnI-R	ggggtaccggcgtgacaagtttcattatgatc
7b-KpnI-F	ggggtaccatgattgaactttcattaattg
7b-XhoI-R	ccctcgagttaggcgtgacaagtttcattatgatc
8-NheI-F	ctagctagcgccaccatgaaatttcttgttttcttagg
8-KpnI-R	ggggtaccgatgaaatctaaaacaacacgaacgtc
8-KpnI-F	ggggtaccatgaaatttcttgttttcttagg
8-XhoI-R	ccctcgagttagatgaaatctaaaacaacacgaacgtc
10-NheI-F	ctagctagcgccaccatgggctatataaacgttttcgcttttc
10-KpnI-R	ggggtacctgtgagattaaagttaactacatctac
10-KpnI-F	ggggtaccatgggctatataaacgttttcgcttttc
10-XhoI-R	ccctcgagctatgtgagattaaagttaactacatctac
N(1-246)-KpnI-R	ggggtaccgacagtttggccttgttgttgttggcctttacc
N(247-419)-NheI-F	ctagctagcgccaccatgactaagaaatctgctgctgaggcttctaag
N(1-246)-NotI-R	atttgcggccgcttagacagtttggccttgttgttgttggcc
N(247-419)-BamHI-F	cgggatccatgactaagaaatctgctgctgaggcttctaag
N-1-R	actcccgccacctccactcccgccacctccggcctgagttgagtcagcactgctcatgg
iRN123-2-F	ggaggtggcgggagtggaggtggcgggagtatggctgaaggatccgtcgccaggc
iRN123-KpnI-R	ggggtaccttaccgctggggaggctcgagctcgagg
PS576-KpnI-F	ggggtaccgagctttgggctaagcgcaacattaaacc
PS576-BamHI-R	cgggatccaccacctaactgactatgactaaaatctcc
Flag-E-NheI-F	ctagctagcgccaccatggattataaagatgacgacgataaaatgtactcattcg
Flag-E-F	atggattataaagatgacgacgataaaatgtactcattcgtttcggaagagacaggtac
E-R	ccacgtcaccgcatgttagaagacttcctctgccctcgaccagaagatcaggaactc
M-F	tcttctaacatgcggtgacgtggaggagaatcccggccctatggcagattccaacggta
Flag-M-KpnI-R	ggggtaccttatttatcgtcgtcatctttataatcctgtacaagcaaagcaatattgtc

Flag-M-1-R	ctctttatcgtcgtcatctttataatcctgtacaagcaaagcaatattgtcactgctac
Flag-M-2-R	tcaccgcatgttagaagacttcctctgccctctttatcgtcgtcatctttataatcctg
N-1-F	atgcggtgacgtggaggagaatcccggccctatgtctgataatggaccccaaaatcag
N-2-F	gagggcagaggaagtcttctaacatgcggtgacgtggaggagaatcccgg
N-3-F	aagatgacgacgataaagagggcagaggaagtcttctaacatgcggtg
N-KpnI-R	ggggtaccttaggcctgagttgagtcagcactgctcatggattgttgc
EGFP-F	atggtgagcaagggcgaggagctgttc
EGFP-R	cttgtacagctcgtccatgccgagagtg

Forward primer; R, Reverse primer
